# Editorial: Recent advances in immunometabolism

**DOI:** 10.3389/fphar.2024.1422816

**Published:** 2024-06-20

**Authors:** Ishtiaq Jeelani, Allah Nawaz, Hafiz Muhammad Asif, Ishtiaq Ahmad, Arijeet K. Gattu

**Affiliations:** ^1^ Division of Endocrinology and Metabolism, Department of Medicine, School of Medicine, University of California, San Diego, San Diego, CA, United States; ^2^ Section on Integrated Physiology and Metabolism, Joslin Diabetes Center, Harvard Medical School, Boston, MA, United States; ^3^ University College of Conventional Medicine, Faculty of Medicine and Allied Health Sciences, The Islamia University of Bahawalpur, Bahawalpur, Pakistan; ^4^ Department of Global Health Research, Graduate School of Medicine, Juntendo University, Tokyo, Japan; ^5^ Metabolism Unit, Massachusetts General Hospital, Harvard Medical School, Boston, MA, United States

**Keywords:** immunce cells, macrophages, metabolism, immuno-metabolic crosstalk, immunomodulatory compounds

## Introduction

Immunometabolism, at the intersection of immunology and metabolism, has emerged as a dynamic field reshaping our understanding of disease mechanisms and therapeutic strategies. This growing area reveals the complex interplay between immune responses and metabolic pathways, with profound implications for health and disease. Recent breakthroughs have elucidated how immune cells modulate metabolic processes and *vice versa*. From interpreting macrophage polarization in obesity-related inflammation to unraveling T cell metabolism in cancer immunity, immunometabolism research has unveiled new insights into disease pathogenesis and therapeutic targets (Nawaz et al., 2021; Kloc et al., 2023; Nawaz et al., 2023). As we get on this journey through immunometabolism, we aim to explore the path forward in understanding the complexities of immune-metabolic crosstalk and elucidating its therapeutic potential.

The study by Li et.al. demonstrates that chronic low-grade inflammation characterizing adipose tissue in obesity serves as a pivotal link to metabolic disorders, primarily planned by adipose tissue macrophages (ATMs). Oleanolic acid (OA), a natural triterpenoid known for its anti-diabetic and anti-inflammatory properties, is a promising candidate for modulating inflammation. However, the underlying mechanisms remain unknown. This study elucidated the intricate pathways through which OA alleviates adipose tissue inflammation in obese mice. C57BL/6J mice subjected to a high-fat diet (HFD) for 12 weeks were administered daily doses of 25, and 50 mg/kg OA for 4 weeks. Compared to vehicle-treated mice, OA administration markedly ameliorated insulin resistance, reduced adipose tissue hypertrophy, and decreased ATM infiltration, thereby rebalancing the M1/M2 macrophage ratio. Notably, OA downregulated pro-inflammatory markers both in adipose tissue of obese mice and in IFN-γ/LPS-treated RAW264.7 macrophages. Mechanistically, OA suppressed mitogen-activated protein kinase (MAPK) signaling and NLRP3 inflammasome activation by targeting voltage-dependent anion channels (VDAC) expression and reactive oxygen species (ROS) production within mitochondria. This study unveils the pivotal role of OA in modulating mitochondrial function and macrophage activation, offering novel insights into its therapeutic benefits in amelioration of obesity-associated adipose tissue inflammation.

The study by Nazir et al. shows that in an arthritic rat model, Campesterol Ester Derivatives (CED) exhibit remarkable anti-inflammatory properties, leading to a significant reduction in paw edema compared to the control group. Histopathological analysis of the treated rats’ supported these findings, revealing a notable decrease in inflammation and tissue damage, characterized by reduced pannus formation and bone erosion. Notably, CED treatment demonstrated a favorable safety profile, with no signs of hepatotoxicity or nephrotoxicity in rats. At the molecular level, CED treatment elicited a downregulation of mRNA expression levels of pro-inflammatory markers, underscoring its capacity to suppress inflammation. Conversely, certain anti-inflammatory markers upregulated following CED treatment, suggesting a transition of cytokines from pro-inflammatory to anti-inflammatory cytokines. Moreover, the beneficial effects of CED extended beyond joint inflammation, as evidenced by its systemic impact on hematological and biochemical parameters. These findings shed light on the multifaceted therapeutic potential of CED in arthritis.

The review article by Otunla et al. discusses the role of dysregulated lipid metabolism in the pathogenesis of various kidney diseases and explores the role of lipotoxicity in acute kidney injury (AKI). In this review, authors summarize the cardinal features of lipotoxic injury in ischemic kidney injury, focusing on lipid accumulation and mitochondrial dysfunction in proximal tubular epithelial cells in ischemic AKI. Additionally, they introduce a novel concept of “immunometabolic” lipotoxicity, shedding light on the intricate interplay between lipid metabolism and immune pathways in AKI pathophysiology. Ultimately, the authors underscore the dual role of lipotoxicity in acting both directly through mitochondrial dysfunction-induced tubule cell apoptosis and indirectly through innate immune system-mediated inflammation. The concept of immunometabolic lipotoxicity represents a paradigm shift in our understanding of AKI pathogenesis, thus suggesting the potential of lipid-lowering therapies to mitigate renal injury in human dyslipidemias. Future research efforts must prioritize the characterization of these immunometabolic pathways to advance our understanding of AKI and improve its clinical management.

The study Mahnashi et al. demonstrates that inflammation, a crucial defense mechanism against various infectious agents, has driven the journey for novel anti-inflammatory drugs. In this study, authors explore the pharmacologically important thiazole scaffold to develop potent anti-inflammatory agents. Employing a multi-step synthetic approach, they synthesized seven novel thiazole derivatives (5a–5g). Initial investigations focused on evaluating the *in vitro* anti-inflammatory properties of these compounds through COX-1, COX-2, and 5-LOX enzyme assays. Following confirmation of their potential, the compounds underwent *in vivo* analgesic and anti-inflammatory studies using the hot plate method for analgesia and carrageenan-induced inflammation assay. Their findings revealed that all compounds exhibited potent inhibition of COX-2, surpassing the efficacy of celecoxib with IC50 values ranging from 0.76 to 9.01 μM. Notably, few compounds emerged as selective COX-2 inhibitors, demonstrating the lowest IC50 values and notable selectivity indices (SI) of 42, 112, and 124, respectively. Although relatively less potent in COX-1 inhibition compared to aspirin, their compounds exhibited encouraging results. Additionally, these compounds exhibited notable efficacy in mitigating inflammation induced by various phlogistic agents and demonstrated significant anti-nociceptive properties. Collectively, the author’s findings underscore the therapeutic promise of thiazole derivatives as effective anti-inflammatory agents, paving the way for further exploration of anti-inflammatory agents.

The review article by Kado et al. discusses the recent advancements in research methodologies, particularly in single-cell-level analysis techniques like single-nucleus RNA sequencing (snRNA-seq) and spatial transcriptomics, which have revealed the intricate diversity within macrophage populations. However, despite gaining clarity on the genetic heterogeneity of macrophages, their functional diversity remains inadequately understood. To bridge this gap, future studies must prioritize the identification of specific markers delineating distinct macrophage subsets, coupled with leveraging gene editing technologies. This approach is imperative for unraveling the functional characteristics of macrophages comprehensively. Furthermore, cutting-edge investigations are poised to delve into several areas, including elucidating macrophage interactions with other cellular components, deciphering the aging process of macrophages, unraveling the epigenetic regulation governing macrophage function, probing into macrophage metabolism, and developing strategies for artificial modulation of macrophage behavior.

## Conclusion

Together, these articles in this Research Topic of recent advances in immunometabolism research present novel immunomodulatory compounds affecting metabolism and showcase two comprehensive reviews of key areas within this field. By connecting metabolism and immune functions, these studies offer avenues for a deeper understanding of complex disease mechanisms and explore potential treatments. Overall, these findings highlight the significance of immunometabolism as a dynamic field with profound implications for developing therapeutics.
